# Enhancement of the Load Capacity of High-Energy Laser Monocrystalline Silicon Reflector Based on the Selection of Surface Lattice Defects

**DOI:** 10.3390/ma13184172

**Published:** 2020-09-19

**Authors:** Gang Zhou, Ye Tian, Shuai Xue, Guangqi Zhou, Ci Song, Lin Zhou, Guipeng Tie, Feng Shi, Yongxiang Shen, Zhe Zhu

**Affiliations:** 1 College of Intelligence Science and Technology, National University of Defense Technology, 109 Deya Road, Changsha 410073, Hunan, China; zg2206553079@foxmail.com (G.Z.); shuaixue1991@163.com (S.X.); guangqizhou@foxmail.com (G.Z.); sunicris@163.com (C.S.); zhoulin_cn@sohu.com (L.Z.); tieguipeng@163.com (G.T.); sf.wind@yahoo.com (F.S.); xiangyueni@163.com (Y.S.); zg3402332161@foxmail.com (Z.Z.); 2 Hunan Key Laboratory of Ultra-Precision Machining Technology, Changsha 410073, Hunan, China; 3 Laboratory of Science and Technology on Integrated Logistics Support, National University of Defense Technology, 109 Deya Road, Changsha 410073, Hunan, China

**Keywords:** high-energy laser monocrystalline silicon, lattice defect, deterministic regulation of optical characteristic, molecular dynamics simulation, ion beam figuring

## Abstract

Various defects during the manufacture of a high-energy laser monocrystalline silicon reflector will increase the energy absorption rate of the substrate and worsen the optical properties. Micron-scale or larger manufacturing defects have been inhibited by mechanism study and improvement in technology, but the substrate performance still fails to satisfy the application demand. We focus on the changes in the optical properties affected by nanoscale and Angstrom lattice defects on the surface of monocrystalline silicon and acquire the expected high reflectivity and low absorptivity through deterministic control of its defect state. Based on the first principles, the band structures and optical properties of two typical defect models of monocrystalline silicon—namely, atomic vacancy and lattice dislocation—were analyzed by molecular dynamics simulations. The results showed that the reflectivity of the vacancy defect was higher than that of the dislocation defect, and elevating the proportion of the vacancy defect could improve the performance of the monocrystalline silicon in infrared (IR) band. To verify the results of simulations, the combined Ion Beam Figuring (IBF) and Chemical Mechanical Polishing (CMP) technologies were applied to introduce the vacancy defect and reduce the thickness of defect layer. After the process, the reflectivity of the monocrystalline silicon element increased by 5% in the visible light band and by 12% in the IR band. Finally, in the photothermal absorption test at 1064 nm, the photothermal absorption of the element was reduced by 80.5%. Intense laser usability on the monocrystalline silicon surface was achieved, and the effectiveness and feasibility of deterministic regulation of optical properties were verified. This concept will be widely applied in future high-energy laser system and X-ray reflectors.

## 1. Introduction

As a core optical element in a high-energy layer system, the monocrystalline silicon reflector has been extensively applied in national defense and industry [1,2]. The laser load capacity of this reflector, which works at extreme conditions under continuous irradiation of high-energy laser, is of vital importance to the overall system performance [3,4,5]. The new-generation intense laser system has proposed harsh requirements for the laser load capacity of monocrystalline silicon reflectors, in which the energy absorption rate needs to be lowered from the current 100 ppm to below 30 ppm.

Although the infrared (IR) single photon has low energy, its high-power continuous output time is long. Thus, the energy absorbed by each element under working conditions is extremely high, and the main laser damage patterns include reversible thermal deformation and irreversible thermal gradient stress failure or material fusion but not high-power damage at the UV band. Such damage and deformation are due to the fact that the laser energy is sedimented inside the element as heat. The laser load capacity of the intermediate IR (MIR) element should be measured by its energy absorption rate (element absorbed energy/laser irradiation energy) [6,7,8]. Energy absorption mainly originates from three parts—namely, intrinsic absorption of the material, absorption caused by substrate defects (e.g., scratches, pocks, and contamination), and absorption induced by film defect (e.g., furuncle). The latter can lead to the local ablation of the surface and irreversible physical damage to a great extent. Given that intrinsic absorption cannot be reversed, studies on the reduction of absorption rate in the academe have been performed with the main focus on the repression of manufacturing defects.

To reduce energy absorption, defects with sizes larger than the micrometer scale on the surface/subsurface of monocrystalline intense laser element have been inhibited very well. Technologies such as float polishing and magnetorheological polishing can improve the micrometer-sized surface defects. With reference to wafer Chemical Mechanical Polishing (CMP) technology, ultra-precise grinding, and immersed fairing, various damage precursors (e.g., scratches, pocks, and medium-high frequency errors) can be effectively restrained [2,9,10,11,12]. In addition, surface smoothness will be better than grade I, and zero defect can be even realized on minor-caliber elements. The surface element contamination is also lower than the Energy Dispersive Spectroscopy (EDS) detection limit. For films, Garrett [13] plated a film with ultra-low absorption rate on the surface of a monocrystalline silicon substrate by using improved technology in 2016.

Although micrometer-scale defects have been effectively inhibited, the use demand cannot be satisfied yet. Under the present extreme condition of higher-energy-level laser irradiation, the thermal deformation of a single element will reach over 2 μm after continuously working for 1 min due to 30-ppm absorption rate. In an actual optical path system, the thermal deformation superposed by multiple elements can exceed remarkably the adjustable range of adaptive optical system, seriously impacting the quality of the optical beam.

Many studies have attempted to retrain extrinsic changes of surface silicon atoms to reduce defect-induced absorption. However, for nanoscale and Angstrom scale, the original repression of micrometer defects are not applicable. The structural defects of near-surface atoms have essential differences from scratches, pocks, and so on, because the former is unavoidable. The atoms at several surface layers of even a perfect monocrystalline silicon under ideal vacuum condition will also undergo relaxation and reconstruction to deviate from the ideal state. Under general atmospheric condition, an oxidation layer will be formed on the monocrystalline silicon surface within dozens of seconds [14]. In addition, silicon atoms will form different chemical structures with H, OH-, F-, and SiO_2_^-^ functional groups in different environments and these chemical structures can seriously lead to laser damage [15]. This layer is difficult to completely remove, reducing energy absorption of nanoscale defects should be transformed from the removal and inhibition of defects into the regulation of defect characteristics of near-surface atoms.

Accounting for an extremely small proportion under many nanoscale and microscale defects on the surface, the influence of the lattice structure of several atomic layers (thickness of approximately several nanometers) on the optical properties of the element has not been paid significant attention among researchers in optical material and processing. However, our early-stage study shows that the change in the state of the monocrystalline silicon surface had a significant effect on reflectivity. The electron transition of valance band in the consumption of photon energy of monocrystalline silicon is the most important absorption process in the study of semiconductors [16]. In addition, different lattice defects will affect the optical properties of monocrystalline silicon and its absorption of incident light energy. Therefore, analyzing the influences of the energy band structure on the optical properties and energy absorption from the perspective of lattice defect will be very meaningful to select the lattice defects during machining and improve the load capacity of optical elements.

According to the Tauc relationship [17] obtained through the law of energy conservation for the monocrystalline silicon, the relationship between the absorption coefficient α and the band gap is expressed as follows:

(1) $$\alpha h\nu =A{\left(h\nu -E_{g}\right)}^{n},$$

where $E_{g}$ is the band gap with the unit of eV; constant A depends on the transition type and is inversely proportional to the difference (ΔE) from the conduction band tail state to the valance band edge; hv is the energy of the incident photon, which is given in units of eV; and n is an index, which is assumed to be 2, because the monocrystalline silicon is an indirect band gap semiconductor.

Meanwhile, the relationship between α and reflectivity R is calculated as follows [18]:

(2) $$2\alpha t=\ln[(R_{\max}-R_{\min})/(R-R_{\min})],$$

where t is the sample thickness given in units of cm; $R_{\max}$ is the maximum reflectivity; $R_{\min}$ is the minimum reflectivity; and R is the reflectivity when the incident photon energy is hv.

From Equation (3), the relationship between the absorption coefficient α and transmission *T* is expressed as follows:

(3) $$T=\frac{I}{I_{0}},$$

where I is the transmitted intensity and $I_{0}$ is the incident laser intensity.

(4) $$\alpha t=-\ln(T)=-\ln(\frac{I}{I_{0}}).$$

As α decreases, the reflectivity *R* and transmission on *T* will increase. As scattered light *S* accounts for a low proportion, and the processing technology proposed in this study can guarantee uniform roughness without any additional damage, the scattered light intensity is unchanged by default. With 1 = *T* + *R* + *A* + *S*, reducing α will lessen the absorption of incident energy. For the chemical structural defects on the surface, the optical properties of the monocrystalline silicon reflector are regulated through the deterministic selection of surface lattice defects, and this characteristic is verified ultimately.

In Section 2, a simulation calculation of the influences of two typical lattice defects on reflectivity and absorption coefficient was implemented. Based on the first-principle calculations, two defect models—namely, atomic vacancy and lattice dislocation—of the monocrystalline silicon structure were established. The energy band structure and optical properties of the two lattice defects were calculated through simulation. The direction of defect regulation is acquired theoretically.

In Section 3, sample preparation and experimental design were introduced. To verify the simulation result and obtain the effects of Section 2, a method of processing technology was used to regulate the lattice defects on the material surface according to the simulation results. To further investigate the lattice defects after processing and compare the influence of different defects on load capacity, Ion Beam Figuring (IBF) was employed to conduct the regulatory processing of the lattice defects on the monocrystalline silicon surface.

The regulation effect was tested and verified in Section 4 and Section 5. Compared with those before the defect regulation, the surface reflectivity increased by 12%, while the photothermal weak absorption rate declined by 80.5% at the 1064-nm band.

Overall, the simulation results have good agreement with actual experiment for the optical properties of the processed sample, which makes the simulation models and parameters more credible and provides substantial theoretical basis and implementation for the method of lattice defect regulation.

The high-energy laser usability on the monocrystalline silicon surface was improved, and the effectiveness and feasibility of the deterministic regulation of the optical properties of elements were verified. The underlying concept will bring new enlightenment to the existing design and processing methods of optical elements and facilitate the improvement of the performance of intense laser elements. Moreover, the principle can be extended to the manufacture of special optical elements and microscale and nanoscale optical devices, such as an X-ray reflecting reflector, to acquire desired optical properties.

## 2. Simulation of Relationship between Lattice Defects and Optical Properties

### 2.1. Construction of Defect Simulation

The atomic vacancy and lattice dislocation defects were modeled by using Materials Studio 7.0, and their optical properties were analyzed by following the first principles based on the density function theory (DFT).

In Materials Studio 7.0, the CASTEP (Cambridge Sequential Total Energy Package) module was used for the band structure and optical properties calculation [19]. An ultra-soft pseudo potential was used to delineate the interaction between the valence electrons and ion core. The revised Perdew–Burke–Ernzerh of generalized gradient approximation (GGA-PRBE) was applied as the exchange correlation functional for geometry optimization of the monocrystalline structure [20], with the energy cut-off of the plane wave set at 350 eV. The Broyden–Fletcher–Goldfarb–Shanno (BFGS) algorithm was selected for the optimization and property calculations of the model, with the self-consistent field (SCF) tolerance set to 2.0 × 10^−6^ eV/atom, then, a reciprocal space k-point of 2 × 2 × 1. Spin polarization was not considered in the calculations [21].

The simulation parameters are listed in Table 1.

**Figure 1:**
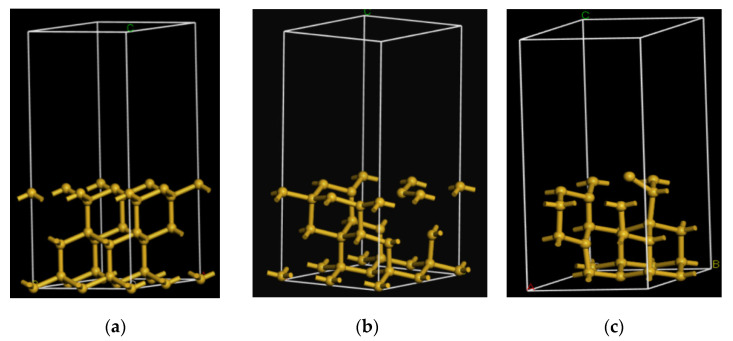
Structural models of (**a**) Defect-free; (**b**) Vacancy; (**c**) Lattice dislocation.

In Materials Studio, a monocrystalline structure was constructed with space group number of 227 and lattice constant of 5.430 A. The primitive cell was periodically extended as 2 × 2 × 1, and a vacuum layer (12 Å-high) was established. The defect-free model A had 34 Si atoms (Figure 1a).

Two Si atoms were removed from the surface structure center in model A, while one Si atom was removed from the middle layer to form model B. Model B had a total of 31 Si atoms (Figure 1b).

Two Si–Si bonds of surface atoms were manually ruptured in model A, and Si atoms were dislocated to form model C (Figure 1c).

Models B and C were simulated by low-temperature annealing, which could eliminate the additional stress induced by manual modeling while guaranteeing the state of atomic layer. The NVE system [22] was selected in the annealing at 750 K. After the annealing was completed, the DISCOVER module was used to minimize the energy of the postannealing model to make the model more approximate to the actual structure. The postannealing and optimized models are shown in Figure 1b,c, respectively. Then, the optical characteristics of the defect models were calculated by simulation.

### 2.2. Simulation Result of Band Structure and Dielectric Function

**Figure 2:**
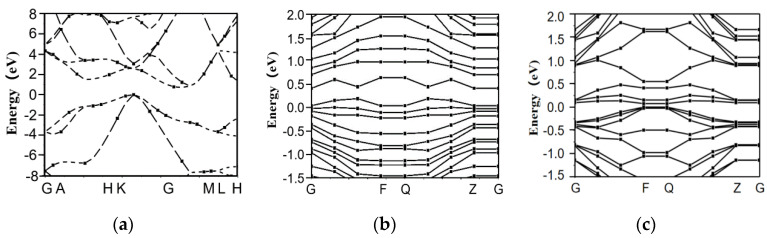
Energy band structures of defect models: (**a**) Defect-free; (**b**) Vacancy model; (**c**) Lattice dislocation.

The band structure reflects the electron transition and photon absorption characteristics. In addition, this structure can be used to analyze qualitatively the reflectivity. The band structures (Figure 2) and optical properties of the introduced lattice defect models were analyzed by simulation.

As shown in Figure 2, (for the x-axis, the G point, A point, and H point, etc., mean the k-points that are selected on the basis of symmetry of simulation model), the band gap of the defect-free model was 0.59 eV (Figure 2a), but the band gap width of Si was *E_g_* = 1.14 eV, which was ascribed to the GGA approximation [23]. However, the analysis will not be impacted by such difference.

The lattice defects resulted in two changes in the energy band structure of the monocrystalline silicon as follows:

(1) The band gap was reduced. The conduction and valance bands evidently moved toward the Fermi level because of the existence of atomic vacancy and dislocation defects. The band gap of the vacancy model became 0.035 eV (Figure 2b), while that of the lattice dislocation model was 0.076 eV (Figure 2c).
(2) The energy level of the monocrystalline silicon was split and became more compact, and new energy levels (defect energy levels) appeared in both defects. The width between the energy levels was narrowed, resulting in easier electron transition between the energy levels [24].

The band structures of vacancy model and lattice dislocation are listed in Table 2.

Although the difference between the two models in the band gap was not large, relative to the dislocation model, the energy level spacing in the vacancy model became more uniform and narrower than the spacing in the energy levels in the dislocation model. The electron transition between energy levels was also easier.

**Figure 3:**
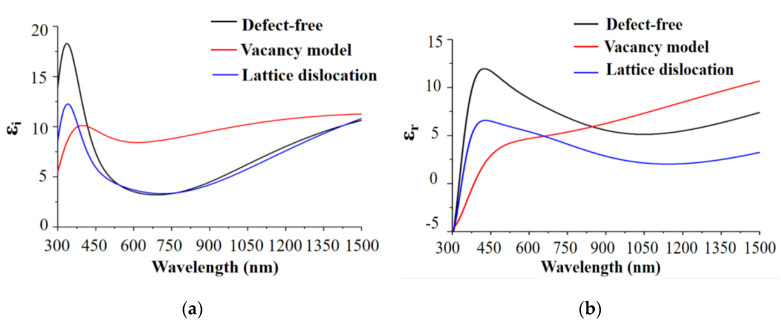
(**a**) Imaginary and (**b**) real parts of the dielectric functions in the different defect models at 300–1500 nm.

The structural change in the energy band caused by the existence of vacancy and lattice dislocation would certainly change the dielectric function. The dielectric functions simulated for the different structures are shown in Figure 3.

The dielectric function reflects the electron transition energy between the conduction and valence bands (Figure 3a). At 500–1500 nm, the imaginary part of the dielectric function of the lattice dislocation model was at the same level as that of the defect-free model. At 500–1500 nm, the imaginary part of dielectric function of vacancy model was obviously greater than that of the defect-free model, because the band gap width of the vacancy defect was narrow. In addition, the probability for electrons to absorb photons became greater, the number of electrons under the excited state was larger, and the electrons could be more easily transited. The next-step reflection would be more probable.

The real part of the dielectric function reflects the material reflection property (Figure 3b). At 500–1500 nm, the dislocation defect model had a smaller real part than the defect-free model. The real part of the vacancy defect was greater than that of the defect-free model after 900 nm.

### 2.3. Simulation Results of Absorption Coefficient and Reflectivity

To further intuitively reflect the optical characteristics of the material, the absorption coefficients and reflectivity of the different lattice defects were calculated through the simulation.

**Figure 4:**
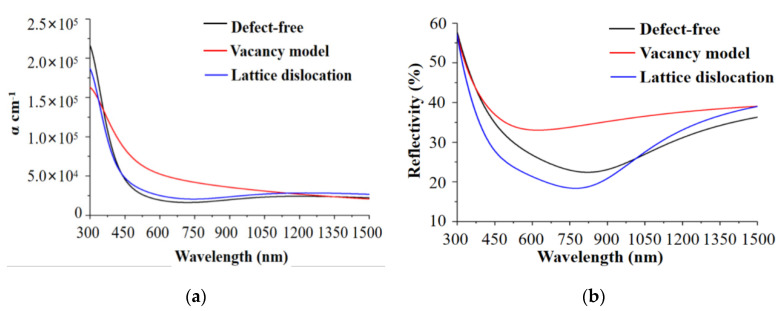
(**a**) Absorption coefficients and (**b**) reflectivity of the defect models at 300–1500 nm.

As shown in Figure 4a, the absorption coefficients of the three models displayed were basically identical variations. The absorption coefficient of the vacancy model was enlarged because of the surface defect at 500–900 nm. This phenomenon was mainly due to the transition of the electron-absorbed energy in the defect energy level toward the conduction band. However, the absorption coefficient of the dislocation model was not remarkably different from that of the defect-free model, because the imaginary parts of the dielectric function of both models were basically at the same levels.

Nevertheless, with the sharp reduction in the band gap width of the vacancy model, the electron transition probability was enlarged and the absorption coefficient was obviously increased. In the area without intrinsic absorption, the absorption coefficient was lowered to the same level as that of the defect-free model.

As shown in Figure 4b, at the wavelength of 500–900 nm (which include a part of visible band), although the absorption coefficient was increased, the surface reflectivity was elevated because of the existence of the vacancy defect. This phenomenon was due to the high photon energy under the small visible-light wavelength. Given the defect energy level and narrowed band gap width, the electrons absorbed lower photon energy so that they could be transited toward the conduction band. Meanwhile, the increasing real part of its dielectric function also enhanced the reflecting property of the surface. Thus, the reflectivity was elevated while the absorption coefficient was increased.

Comparison of the energy band structures and optical properties of the models showed the following characteristics:

(1) Given the change in the energy band structure and the increasing imaginary part of the dielectric function of vacancy defect, the reflectivity of the sample surface was elevated within the waveband of 500–1500 nm.
(2) The reflectivity at 500–900 nm was reduced because of the lattice dislocation, and the reflectivity at over 1000 nm was slightly higher than that of the defect-free model.

The comparison results of reflectivity between three models are listed in Table 3.

Therefore, to acquire a surface with higher reflectivity and smaller absorption coefficient, the surface defect should be regulated to a state with more vacancies and fewer dislocations. Consequently, the reflectivity could be elevated by 3–5% near the working waveband of 1064 nm of the monocrystalline silicon. This characteristic would play a decisive role in the application of intense laser. Then, processing and detection were combined to further explore the effect of deterministic regulation of surface defects.

## 3. Method of Defect Regulation

Deterministic Generation of Vacancy and Dislocation Defect

The two defects were generated by using separately IBF (Ion Beam Figuring) and CMP (Chemical Mechanical Polishing). To acquire high precision and good surface quality in actual processing, IBF and CMP were generally alternated. A sample was also prepared using IBF + CMP process (Combined Process). Thus, a control sample with unregulated defects was developed. The preceding-stage surface treatment for all four samples was CMP. The processing methods and material information of four samples are shown as Table 4.

Sample #1, as an important initial sample, the optical properties of it will offer important reference to us, so, we can gain the preceding-stage processing parameters in Table 5.

As a comparison sample, the #1 sample was only wiped by ethanol and acetone, without any other form of processing.

During the process of CMP, sample #1 was polished for 20 min using a 100-nm SiO_2_ gel polishing agent in the pitch disk as a comparison of the surface optical characteristics under the traditional polishing technology. After CMP, the amorphous layer and slip dislocation were also generated on the surface. However, no low-density area existed—i.e., no vacancy defect was generated [25]. Relative to the initial state, for sample #2, the polishing pressure was increased to ensure the generation of the dislocation defect. The parameters are listed in Table 6.

**Figure 5:**
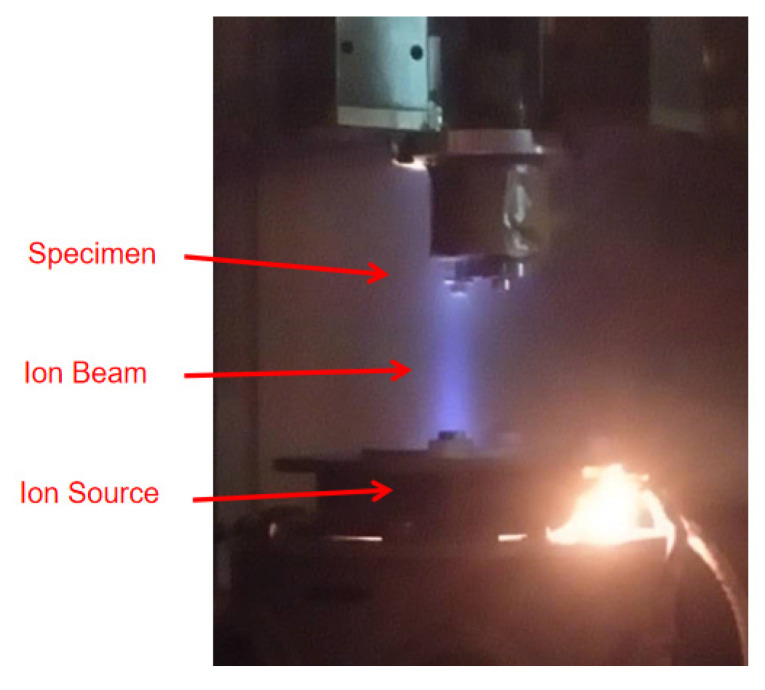
Sample #3 in IBF processing.

The lattice arrangement on the #3 optical element was changed by IBF technology. The ion sputtering effect influenced the lattice arrangement to form an amorphous layer [26]. When the dose of ion beam was very low (<2 × 10^13^ cm^−2^), the ion injection would only generate an isolated Frenkel pair (gap–vacancy pair) or a small defect cluster, i.e., vacancy defects [27]. Meanwhile, as an effective processing method to improve the laser performance [28,29], IBF is usually used to acquire an ultra-smooth surface [30] or is applied to the precoating cleaning of sample surface [31]. IBF was performed on the self-developed KDIBF650L-VT machine (Figure 5) and atomic vacancy defects were introduced. The parameters are listed in Table 7.

Sample #4 was processed by combined CMP and IBF. The processes of sample #1 and sample #3 were iterated on the surface of sample #4 for 4 times, and the total removal amount reached 1.3 μm. Thus, an amorphous layer and atomic vacancy simultaneously existed on the surface. The concrete parameters are shown in Table 8.

**Figure 6:**
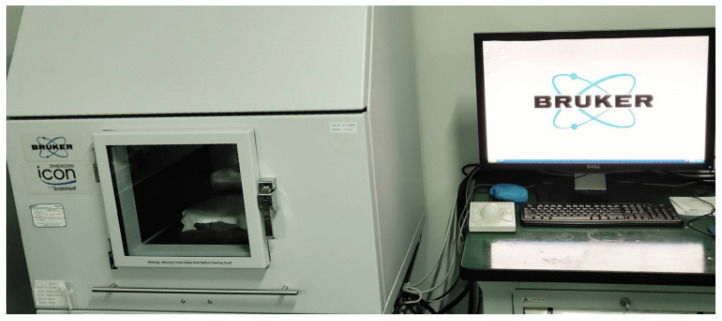
Atomic force microscope (Bruker Dimension Icon). The results showed that the roughness values were small after processing (Figure 7).

The surface roughness of the prepared samples was detected by using an atomic force microscope (AFM, Figure 6) with scanning area of 3 × 3 μm and 512 scanning lines under tapping scanning mode.

## 4. Performance Test Analysis after Defect Regulation

### 4.1. Reflectivity Test

To verify the effectiveness of the regulation of different defects and quantitatively analyze the change in surface reflectivity, the surface reflectivity of the four samples was determined using a spectrophotometer (HITACHI U-4100, HITACHI, Tokyo, Japan).

The test parameters are listed in Table 9.

**Figure 8:**
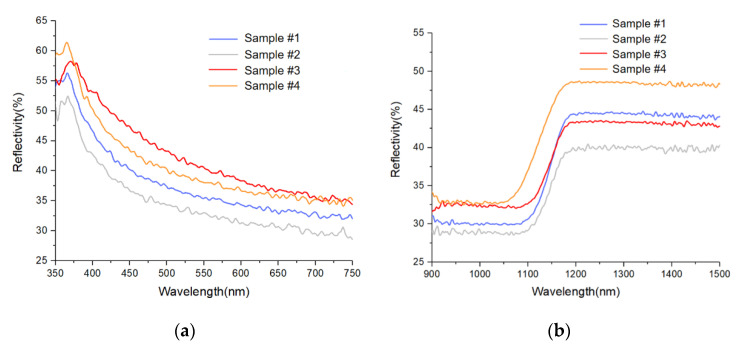
Reflectivity test results: (**a**) Visible-light waveband; (**b**) infrared (IR) band.

The reflectivity test results are shown in Figure 8.

As shown in Figure 8a, the result agreed very well with the simulation result within the visible-light waveband (400–750 nm). The surface reflectivity after the IBF and Combined Process was increased by 5–8%, which was higher than the simulation result of the vacancy model by 3–13%. The simulation results are quite consistent with the values from engineering practice and existing studies.

However, the test result at the IR band reflected various interesting new phenomena as follows (Figure 8b).

**Figure 9:**
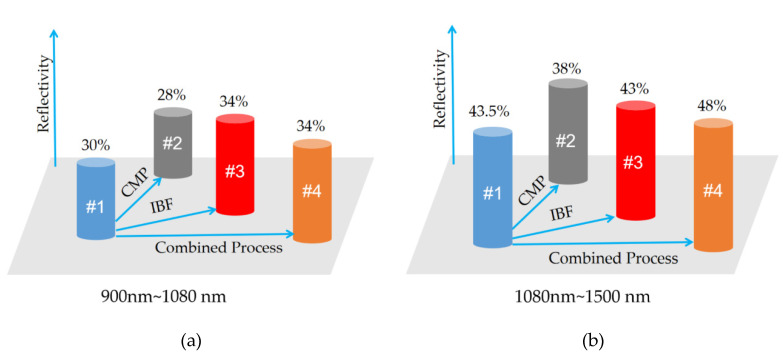
Mean values of the reflectivity of the four samples within (**a**) 900–1080 nm and (**b**) 1080–1500 nm.

(1) The reflectivity sharply increased. Within 1100–1180 nm, the reflectivity values of all four samples evidently increased (Figure 8b), because the intrinsic absorption limit of the monocrystalline silicon was nearly 1110 nm. When the wavelength of incident light was greater than 1110 nm, intrinsic absorption would not be generated, and the reflectivity presented an obvious increasing trend.
(2) The intrinsic absorption limit blue-shifted slightly after the treatment with the combined process.
(3) Within 900–1080 nm, the reflectivity of sample #4 was basically at the same level as that of #3 sample but higher than that of sample #1 by 4% (Figure 9a).
(4) Within 1180–1500 nm, the reflectivity of sample #4 was elevated by 8% compared with that of sample #3. However, the reflectivity of sample #3 was reduced by 2% compared with that of sample #1 (Figure 9b).
(5) The reflectivity on the surface of sample #2 was reduced within the whole frequency band, because the elevated polishing pressure enlarged the thickness of the amorphous layer.

We can see the reflectivity comparison of four samples in Table 10.

### 4.2. Discussion

The four samples under the different surface defect states presented distinct optical characteristics. The optical properties underwent many interesting changes after the defect regulation, and some of these changes were in good agreement with the expected simulation result (Table 11.).

(1) The reflectivity of sample #3 and sample #4 samples relatively increased after atomic vacancy defects were generated through the ion beam process.
(2) The reflectivity was reduced after polishing enlarged the thickness of the amorphous layer (sample #2).

**Figure 10:**
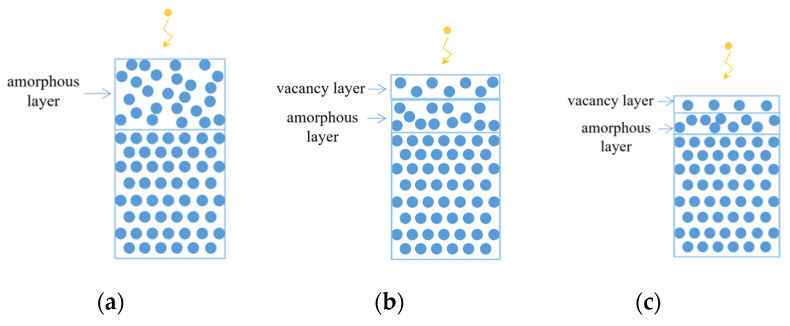
Lattice defect structures on surface of the (**a**) #1, (**b**) #3 and (**c**) #4 samples.

However, some changes deviated from the expectation, as follows:

Phenomenon 1. Although the surface of sample #1 was smooth, the preceding-stage process was CMP polishing. The thickness of amorphous layer was large (Figure 10a). Numerous lattice imperfections were left on the surface, and these defects would enlarge photon absorption and reduce the surface reflectivity in Visible-light waveband, as shown in Figure 8a.

Phenomenon 2. The processing time of sample #3 was short after IBF processing, vacancy imperfections were introduced only at the surface layer, and the surface residual layer could not be totally removed (Figure 10b). Therefore, when the wavelength was smaller than 1150 nm (Figure 8b), the absorption coefficient was large. In addition, the photon propagation distance within the sample was short, and most of the photon energy was absorbed at vacancy layer. The electron transition also accounted for the major part of energy absorption, and the reflectivity was elevated because of the vacancies.

When the wavelength was greater than 1150 nm, no intrinsic absorption existed, and the absorption coefficient was small. In addition, the photon propagation distance inside the sample was long, and most photons entered the amorphous layer. As the energy level became denser because of the defects, the electron transition probability between valence bands increased. Then, numerous excitons and charge carriers were generated to enlarge the absorption of photon energy and lower the reflectivity.

Phenomenon 3. Through multiple iterative processing, the residual layer formed by CMP process on the surface of sample #4 was removed. After polishing, the thickness of the amorphous layer on the surface was small. After the ion beam processing, the thickness of the vacancy layer was large, the atomic density was low, and vacancies were dense (Figure 10c).

Therefore, when the intrinsic absorption accounted for most of the energy absorption at wavelengths smaller than 1150 nm, the absorption mechanism was consistent with that after IBF processing, and their reflectivity values were at the same level. However, when the wavelength became greater than 1150 nm, as the thickness of amorphous layer was small, the absorption of photon energy was also small, and the influence on reflectivity was reduced. The distance of photon propagation inside the sample was long, and most photons entered the lattice layer. Then, the surface vacancy layer played a dominant role in the reflectivity, remarkably elevating the reflectivity.

## 5. Photothermal Weak Absorption Test

**Figure 11:**
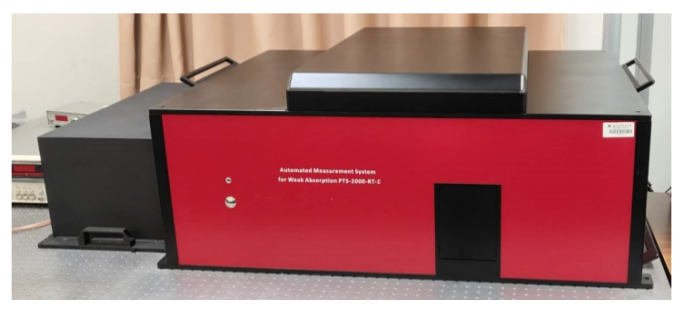
Photothermal weak absorption test platform.

For the continuous pulse high-energy laser, the photothermal absorption has been widely applied to test laser load capacity. Hence, the photothermal absorption test (the testing platform is shown in Figure 11) was performed to evaluate the photothermal absorption characteristics of the samples. This method detects the absorbed energy in the surface microcell of the material by using the deflection effect of the thermal lens. The test result can represent the energy absorption rate of the intense laser element of monocrystalline silicon [32].

The detection parameters are shown in Table 12.

**Figure 12:**
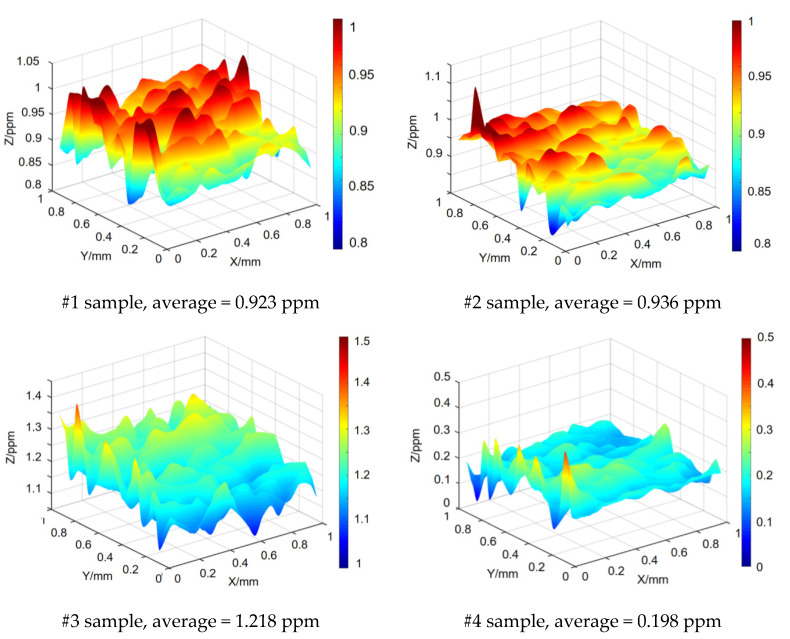
Photothermal weak absorption results of the sample surfaces after different processes (different color indicate the height on Z axis; and ppm, partial per million, means the absorptivity of the irradiation laser energy).

The photothermal weak absorption test was performed on the surfaces of the four samples to evaluate their surface heat absorption levels after treatment. The results are presented in Figure 12.

As shown in Figure 12, a single processing plan (#2, #3) had minor influence on the photothermal weak absorption value. The minimum photothermal weak absorption was observed in sample #4. Compared with the #1 sample, the photothermal weak absorption of sample #2 slightly changed, while that of sample #3 slightly increased.

Given that the pump light wavelength was 1064 nm, the absorption coefficient of model B was slightly greater than those of models C and A (Figure 4a). Meanwhile, the amorphous layer on the surface before the sample treatment enlarged the absorption of photon energy and, consequently, the surface reflectivity was only elevated by 3% after ion-beam processing. Hence, the photothermal absorption value of sample #3 was increased slightly.

The amorphous layers on the surface of the sample #4 were lessened after processing, reducing the absorption of photon energy by the amorphous layers. At 1064 nm, when the difference in absorption coefficient was not large, the surface reflectivity was elevated by 10% because of the vacancy defects (Figure 4b). In addition, the photothermal weak absorption value was greatly reduced to 19.4% of that under the initial stage. The photothermal weak absorption result was identical with the spectral analysis result. Thus, the effectiveness of the simulation analysis and defect regulation was verified.

## 6. Conclusions

Through the simulation calculation of structural defect characteristics, defect regulation, and absorption test, the optical characteristics and energy absorption of monocrystalline silicon reflector could be effectively controlled by regulating the chemical structural defects on the reflector surface.

First, the band structures and optical properties of two typical defect models—namely, atomic vacancy and lattice dislocation—of the monocrystalline silicon were calculated based on the first principles. The reflectivity of the vacancy defect was higher than that of the dislocation defect, and elevating the proportion of vacancy defect could improve the performance of monocrystalline silicon in the IR band. After the vacancy defects were generated and the thickness of defect layer was reduced by the combined process (i.e., IBF and CMP), the surface reflectivity of the monocrystalline silicon element was regulated and elevated by 5% in the visible-light band and by 12% in the IR band. In the final photothermal absorption test, the photothermal absorption value of the element was reduced by 80.5%. Hence, the usability of intense laser on monocrystalline silicon reflector was improved on the small-caliber test samples.

Moreover, the deterministic regulation of the surface defect was preliminarily verified. In the follow-up study, regulation methods can be established for more applications by investigating the different defect characteristics, process-defect correlations, and material adaptability. In addition, this study also provides a new concept to design and process optical materials, that is, to acquire uniformly predictable, designable, and tunable microcell optical characteristics or according to a specific area on the smooth substrate surface of the element.

This principle will bring new enlightenment to the existing design and processing of optical elements and facilitate the improvement of the performance of intense laser elements. Furthermore, in special optical elements and micro- and nanoscale optical devices, such as an X-ray reflector, the method of lattice defect regulating will take the thinking of manufacture into a new dimension. For MOS (Metal Oxide Semiconductor) devices, the IBF technology will change the status of chemical bond on the Si/SiO_2_ interface to obtain better optoelectronic properties, and the principle of changing the reflectivity by the method of defect regulation will provide a potential approach to make the monocrystalline silicon solar cell convert more solar energy. In summary, the principle can also be extended to guide the manufacturing process and acquire the desired optical characteristics.

## Figures and Tables

**Figure 7:**
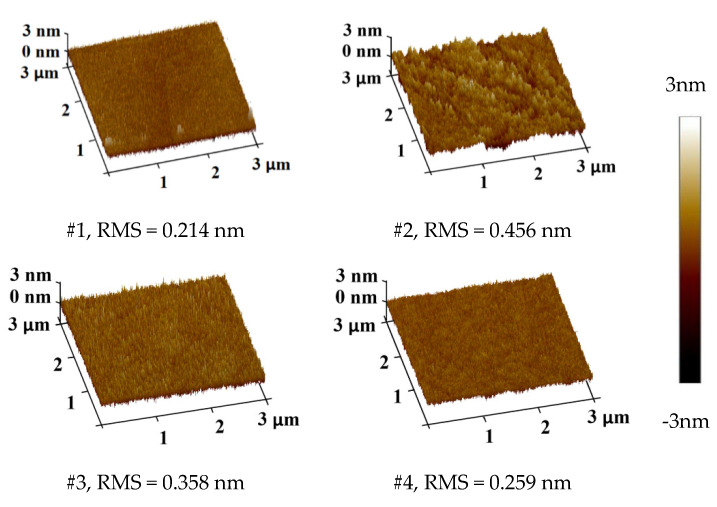
Surface roughness values of the post-treatment samples.

**Table 1:** Simulation parameters. GGA-PRBE: Perdew–Burke–Ernzerh of generalized gradient approximation.

Item	Level
Pseudopotential	Ultra-soft
Functionality of exchange energy	GGA-PRBE
Self-consistent convergence tolerance	2 × 10^−6^ eV/atom
Energy cutoff	350 eV
k-point	2 × 2 × 1

**Table 2:** Band structure of vacancy model and lattice dislocation.

Item	Variation	
	Vacancy Model	Lattice Dislocation
Band gap	Reduced to 0.035 eV	Reduced to 0.076 eV
Energy level	Split More compact Defect energy levels appeared	Split More compact Defect energy levels appeared
Electron transition probability	Higher than Defect-free	Higher than Defect-free

**Table 3:** Comparison of the reflectivity of three models

Wavelength (nm)	Reflectivity
500–900	Vacancy defect > Defect free > Lattice dislocation
1000–1500	Vacancy defect > Lattice dislocation > Defect free

**Table 4:** Sample number and treatment process. CMP—Chemical Mechanical Polishing; IBF—Ion Beam Figuring.

No.	Processing Method	Crystal Orientation	Thickness (mm)	Size (mm)
1	Nontreated	(100)	5	Φ20
2	CMP	(100)	5	Φ20
3	IBF	(100)	5	Φ20
4	Combined Process	(100)	5	Φ20

**Table 5:** Preceding-stage processing parameters (#1).

Item	Level
Pressure	0.01 MPa
Polishing agent	Nano SiO_2_ gel (100 nm, pH 9)
Polishing disk	Pitch polishing disk

**Table 6:** CMP parameters (#2).

Item	Level
Pressure	0.03 MPa
Processing time	20 min
Polishing agent	Nano SiO_2_ gel (100 nm, pH 9)
Polishing disk	Asphalt polishing disk
Removal amount	85 nm

**Table 7:** IBF parameters (#3).

Item	Level
Voltage	900 eV
Sputtering gas	Argon
Base pressure of the chamber	1.5 × 10^−3^ Pa
Incidence angle	0°
Processing time	30 min
Processing mode	Uniform Scanning

**Table 8:** Parameters of Combined Process (#4).

Number of Times	Processing Method	Processing Time	Process Parameters	Removal Amount
1, 2	CMP	30 min	Polishing pressure: 0.03 MPa Polishing agent: SiO2 gel (100 nm, pH 9) Polishing disk: asphalt polishing disk	300 nm
	IBF	30 min	Voltage (eV): 900 eV Incidence angle: 0° Processing mode: Uniform Scanning	120 nm
3, 4	CMP	15 min	Polishing pressure: 0.02 MPa Polishing agent: SiO2 gel (50 nm, pH 9) Polishing disk: asphalt polishing disk	120 nm
	IBF	20 min	Voltage: 900 eV Incidence angle: 0° Processing mode: Uniform Scanning	85 nm

**Table 9:** Spectrophotometer parameters.

Item	Level
Angle	15°
Polarization	S
Measuring	15350–1500
waveband	nm

**Table 10:** Comparison of the reflectivity of four samples.

Wavelength (nm)	Reflectivity
900–1080	#3>#4>#1>#2
1080–1180	The reflectivity evidently increased
1180–1500	#4>#1>#3>#2

**Table 11:** The reflectivity variation of three samples contrast to sample #1.

**Sample**	**Evolution of Surface**	**Variation of Reflectivity**
#2	Thickness of amorphous layer was enlarged	Reduced
#3	Atomic vacancy defects were generated	Increased
#4	Atomic vacancy defects were generated	Increased

**Table 12:** Detection parameters.

Item	Level
Irradiation laser wavelength	1064 nm
Irradiation laser power	4 watt
Scan size per test	1 mm × 1 mm
Pixel size	50 μm × 50 μm
Test mode	reflective
